# B-Mode Ultrasound Diagnostics of Orthopaedic Diseases in Clinical Avian Medicine: Comparative Study

**DOI:** 10.3390/ani16101439

**Published:** 2026-05-08

**Authors:** Anna Korshunova, Volker Schmidt

**Affiliations:** Clinic for Birds and Reptiles, Veterinary Faculty, University of Leipzig, An den Tierkliniken 17, 04103 Leipzig, Germany

**Keywords:** bird medicine, diagnostic imaging, ultrasound, musculoskeletal system

## Abstract

Orthopaedic disorders represent a significant clinical challenge in avian medicine, affecting both pet and wild birds. B-mode ultrasound (US) examination is well established in small-animal and equine medicine; it has seen limited application in avian medicine thus far. This study aimed to assess the diagnostic efficacy of B-mode US in comparison to radiography (RX) for orthopaedic avian disorders. A total of 55 birds from six orders were assessed clinically, radiologically, and sonographically. Statistical analysis was conducted for the overall cohort (*n* = 55) and for a fracture subgroup (*n* = 51). Cohen’s kappa was used to examine diagnostic agreement. Sensitivity, specificity, and positive and negative predictive values were also computed. Additionally, Fisher’s exact test was employed to evaluate the representativeness and interpretability of both techniques. In the overall cohort, US demonstrated higher sensitivity (97.6%) and specificity (100%) than RX (sensitivity 70.7%, specificity 85.7%). In the fracture subgroup, US demonstrated superior sensitivity (97.3%) compared with RX (75.7%) while maintaining high specificity (100%). The agreement between imaging findings and the clinical reference standard was substantial to good (κ = 0.64 for the total cohort, *p* < 0.05; κ = 0.74 for the fracture subgroup, *p* < 0.05). No significant differences were identified between the two modalities in terms of representability and interpretability. However, these findings should be interpreted with caution, as US examinations were performed following RX assessment on a non-blinded basis, which may have introduced observational bias. Despite these limitations, B-mode US appears to provide valuable additional diagnostic information and may serve as a complementary imaging modality in the evaluation of avian orthopaedic conditions. In particular, it may be useful for assessing shoulder girdle injuries, inflammatory conditions of the shoulder joint, and fractures of the sternum and keel, as well as for monitoring bone healing following surgical or conservative treatment.

## Simple Summary

In avian medicine, orthopaedic issues are frequently documented in both domesticated and wild birds. Orthopaedic disorders are common in both pet and wild birds and represent a significant challenge in avian medicine. Although radiography (RX) is the primary imaging modality used to assess the avian musculoskeletal (MSK) system, particularly for fracture evaluation, its ability to visualise soft tissues and subtle structural changes is limited. Advanced imaging techniques such as computed tomography (CT) are available but are not routinely used due to their cost and limited accessibility. B-mode ultrasonography (US) is widely used in small-animal and equine medicine; however, its use in avian patients has so far been limited. In this study, we compared US and RX in birds with suspected orthopaedic conditions and analysed their diagnostic performances. Overall, this study highlights the potential role of B-mode US as a complementary imaging technique in avian orthopaedics and provides a basis for its further application in clinical practice.

## 1. Introduction

Orthopaedic problems are frequently reported in pet and wild birds within avian medicine [1]. Environmental factors, including urban infrastructure and restricted habitats in metropolitan and surrounding regions, can facilitate the incidence of trauma in wild avian species. A study by Wendell et al. (2002) indicates that trauma-related disorders constitute 66.3% of the mortality and morbidity causes in wild birds in the USA [2]. Nutritional deficits and stress significantly contribute to the development of metabolic and degenerative disorders in avian species [3]. Scientific research indicates that insufficient UVB exposure and mineral deficiencies during winter may facilitate the development of metabolic bone disease (MBD) in juvenile birds. Cousquer et al. report that the overall prevalence of MBD in wild adolescent wood pigeons is 51.2% [4]. The rehabilitation of wild birds in a clinical setting is frequently a complex endeavour, with its effectiveness, extent, and results contingent upon the initial reason for admission and varying per avian group. There is an urgent need for sophisticated techniques to evaluate the survival rates of patients reintroduced into the wild [5]. Further research is essential for the proper implementation of diagnosis and treatment in these instances.

Radiographic (RX) examination is currently the primary technique for diagnosing musculoskeletal (MSK) diseases in avians [6,7]. This technique has proven effective in detecting fractures and conducting preliminary diagnostic assessments, providing essential information about bone structure and enabling a comprehensive examination of the entire anatomy of avian patients. Nonetheless, superimpositions, particularly in the shoulder girdle and pelvic region, may compromise the clarity of features and structures [8]. Visser et al. (2015) introduce an alternative radiography perspective (H-view) that may enhance the visualisation of shoulder girdle tissues in some instances [9]. The RX evaluation of external soft tissues and minor bone diseases in avians is constrained, limiting diagnostic options for the MSK system [7,10,11,12]. Anatomical superimpositions, the relatively small size of many avian species, and the limited differentiation of soft tissue structures can hinder the interpretation of radiological findings and impede the identification of subtle osseous lesions [11,13,14]. Moreover, evaluation for fissure fractures can be especially complex in avian and exotic animals because their skeletal structures are relatively small and thin, necessitating the use of a magnifying glass and careful scrutiny of the cortical margins adjacent to the fracture site on orthogonal radiographic views [11,13].

The application of computed tomography (CT) in avian medicine is well documented and demonstrates markedly enhanced sensitivity for detecting lesions in intricate anatomical areas, including the shoulder girdle, sternum, and keel, as well as soft-tissue pathologies involving muscles, ligaments, tendons, and cartilage [6,7,10,15]. This imaging technology is typically associated with substantial technological demands and costs, thereby considerably restricting its use in avian medicine, particularly in wild birds [10].

Sonographic evaluations are widely used in mammalian and human orthopaedics, facilitating visualisation of disease in osseous and soft-tissue structures [13,16,17,18,19,20,21,22]. Kramer et al. (1997) utilise ultrasonography (US) using a 7.5 MHz linear transducer or a 5 MHz convex transducer to visualise tendons, muscles, joint effusion, capsular thickening, and tissue ruptures in dogs and cats [18]. Nevertheless, it has been found that the joint surfaces in this area are frequently not entirely accessible. US is used to evaluate soft tissue swellings, muscles, tendons, and joints in exotic animals. The application of colour and power Doppler US is instrumental in evaluating blood flow in certain organs or lesions, assessing ischaemia, or quantifying vascularisation in a mass [10]. US is presently examined in a limited number of studies within avian orthopaedics. González et al. (2018) elucidate the application of the B-mode in evaluating long bones across diverse species of owls and raptors [14]. The study also offers insights into the efficacy of B-mode US for evaluating fractures in live avians and includes a case study on the real-time sonographic observation of surgical stabilisation of bone fragments in a barn owl (Tyto furcata) [14]. Our prior research demonstrated that B-mode US is an efficient technique for evaluating the entire MSK system in live urban pigeons, particularly in the thoracic region and the proximal segments of the limbs. This approach enables the measurement of structures exceeding 0.2 cm in thickness, which is pertinent for planning osteosyntheses or monitoring neoplastic alterations in the avian MSK system [23].

This study aimed to evaluate the effectiveness and suitability of B-mode US for diagnosing specific orthopaedic disorders in avian species. Therefore, specific pathologies in the avian MSK system were analysed using RX and US, followed by a statistical evaluation and comparison of the efficacy of both imaging modalities. The primary objective of this study was to create a theoretical foundation for B-mode US examination to enable its prospective application in clinical practice.

## 2. Materials and Methods

### 2.1. Sample Selection

The study involved 55 birds of various species between 2022 and 2024. The birds were referred to the Clinic for Birds and Reptiles at Leipzig University with clinical signs of MSK lesions (Table 1).

The birds presented varied in terms of age, gender, physical condition, and origin, as well as body measurements (Table 2).

### 2.2. Physical Examination and Clinical Assessment

All birds underwent a standardised clinical assessment, including documentation of their history, initial distance observation, and general and systematic clinical examination [24]. Further diagnostic imaging was initiated based on the birds’ origin, the severity of their injuries, and the favourable prognosis that followed, particularly in the case of migratory birds, regarding their ability to reintegrate into the wild.

### 2.3. Radiographic Assessment

The RX examination utilised a Gierth HF200 X-ray machine (Gierth X-Ray International GmbH, Riese, Germany), Fuji Film radiological detector software (DR-ID 300CL APL Software V14.0.0017, FUJIFILM Corporation, Tokyo, Japan), and IntelliSpace Radiology 4.7 imaging software (IntelliSpace Radiology, Philips, Amsterdam, The Netherlands), and was conducted while the birds were conscious, with a duration of 2 to 3 min per bird. The radiological machine settings varied according to the body weight (40–54 kV, 0.5–5 mAs, 0.04 s). Images of each subject were captured in at least two projections: ventrodorsal and laterolateral. The bird was positioned in dorsal recumbency, with its hind legs and wings extended, utilising an RX positioning apparatus for avians (Polnet Sp. z.o.o., Tarnowo Podgórne, Poland) and lead weights. In the laterolateral projection, the patient was positioned in right lateral recumbency, with the wings extended dorsally and the hind limbs extended caudally. In birds with suspected wing-region injuries, an additional caudocranial image of the extended wing was obtained. An oblique image (H-view) was obtained for individuals suspected of having an injury in the shoulder girdle region. During this examination, the avian specimen was placed supine, and the area of interest was scanned at a 45-degree angle. Each bird was restrained using a plastic holder and lead weights.

### 2.4. Ultrasonographic Assessment

The US examination was conducted using a LOGIQ S7 Expert ultrasound scanner (General Electric [GE], GE Healthcare, Buckinghamshire, UK) in conjunction with a GE L8-18I-D ‘hockey stick’ transducer (AME Ultrasound, Suffern, NY, USA, 8–18 MHz frequency) while the birds were conscious and lasted between 2 and 5 min per bird. Each bird was restrained by an assistant manually in an upright position, as described in our prior study [23]. The examination was conducted methodically, with consideration of the suspected diagnoses derived from medical history, clinical assessments, and radiographic evaluations. The plumage in the examination area was dampened with an alcohol-based disinfectant spray (B.Braun Softaspray^®^, B. Braun SE, Melsungen, Germany) and clipped to one side, after which US gel (Covidien Aquasonic^®^ Ultrasound Gel, Parker Laboratories, Inc., Fairfield, NJ, USA) was administered to the skin. The sonographic approaches outlined in our prior study were employed to evaluate the anatomical structures [23]. The US images of the altered structures were preserved and recorded.

### 2.5. Post-Treatment Bone Healing Monitoring

Four birds [feral pigeon (*Columba livia* f.dom), *n* = 3; common kestrel (*Falco tinnunculus*), *n* = 1] received surgical fracture treatment (three humeral fractures and one femoral fracture), while two feral pigeons with an ulnar and humeral fracture were treated conservatively using an eight-turn splint [25] and analgesics (Metacam^®^, Boehringer Ingelheim, Ingelheim am Rhein, Germany; meloxicam 0.5 mg/kg PO BID for 3–5 days). For the surgical treatment, the ESF IMP tie-in technique was used [26]. Before the surgical intervention, butorphanol (Butorgenic^®^, MSD Animal Health, Kenilworth, NJ, USA) was supplied intramuscularly at a dosage of 2 mg/kg 15 min prior, and meloxicam (Metacam^®^, Boehringer Ingelheim, Ingelheim am Rhein, Germany) was administered subcutaneously at a dosage of 0.5 mg/kg BID. Subsequently, inhalation anaesthesia was administered using isoflurane (Isofluran CP^®^, 1 mL/mL, CP-Pharma GmbH, Burgdorf, Germany) at 5% during induction and 2–2.5% during maintenance. The birds were observed and treated daily following surgery in an inpatient setting. Follow-up evaluations to determine callus formation were conducted on days 7 and 14 post-operatively, using RX and US modalities. Four birds (two humeral fractures, an ulnar fracture, and a femoral fracture) exhibited effective fracture healing. In three birds, the ESF IMP tie-in fixation was excised, and in one bird, the figure-of-eight bandage was detached after 14 days, prior to their successful release into the wild.

### 2.6. Data Analysis and Statistical Evaluation

All examinations, including clinical, radiographic and morphological assessments, were carried out on all birds by an experienced clinician. For the statistical analysis, two case groups were established: a total group (*n* = 55), which included all birds with MSK impairment (*n* = 41) as well as birds with no identified pathologies (*n* = 14); and a fractures subgroup (*n* = 51), which comprised birds with fractures (*n* = 34), including fractures monitored following surgical intervention (*n* = 6), pathological fractures (*n* = 3) and birds without pathologies (*n* = 14).

Data on bird species, sex, body weight, medical history, clinical signs, final diagnosis, and findings from the diagnostic procedures (radiography and ultrasonography) were systematically recorded in an Excel spreadsheet. Four-field contingency tables were constructed to descriptively assess the agreement between each imaging modality and the overall clinical assessment. The latter was used as an internal reference standard for this study, based on the combined evaluation of clinical findings and diagnostic results, as well as clinical follow-up and therapy response. Based on the four-field tables, the diagnostic quality parameters of sensitivity, specificity, and positive and negative predictive values were calculated for both imaging modalities. The corresponding parameters were determined separately for both the overall group and the fracture group. Cohen’s Kappa was calculated to assess the agreement between the respective imaging modalities (X-ray or ultrasound) and the clinical yes/no diagnosis. The strength of agreement was interpreted according to the classifications by Altman et al. (1991) and Landis et al. (1977) [27,28]. A comparison of representability and interpretability between radiography and ultrasound was conducted. Fisher’s exact test was employed due to the categorical nature of the data and the occasionally limited sample sizes. The analysis was conducted independently for the general cohort and the fracture subgroup. The significance level was established at α = 0.05 for all statistical analyses. The statistical analysis was conducted using Microsoft Excel (Version 2024, Microsoft Corporation, Redmond, WA, USA) and SPSS (Version 2025, IBM Corp., Armonk, NY, USA).

## 3. Results

A total of 100 X-rays and approximately 250 ultrasound recordings of 16 different pathologies were produced (Table 3). The optimal US image quality in birds weighing between 130 and 3000 g was subjectively estimated to be delivered by a transducer frequency setting of 15 MHz.

US identified 27 fracture cases, whereas RX detected 24. The observed difference was associated with fractures that were not clearly visualised on radiographs, including lesions of the clavicle (*n* = 2), scapula (*n* = 1), and elbow joint (*n* = 1). In addition, two pathological tibiotarsal fractures were not definitively identified on radiographic examination. In the intact subgroup, ultrasonography classified 14 cases as normal, compared to 12 cases identified by radiography. This discrepancy was related to two cases (scapula, *n* = 1; clavicle, *n* = 1) that were interpreted as abnormal on radiographs but showed no pathological findings on ultrasonographic examination and clinical reference assessment (Table 3).

### 3.1. Shoulder Girdle and Sternum

**Figure 1:**
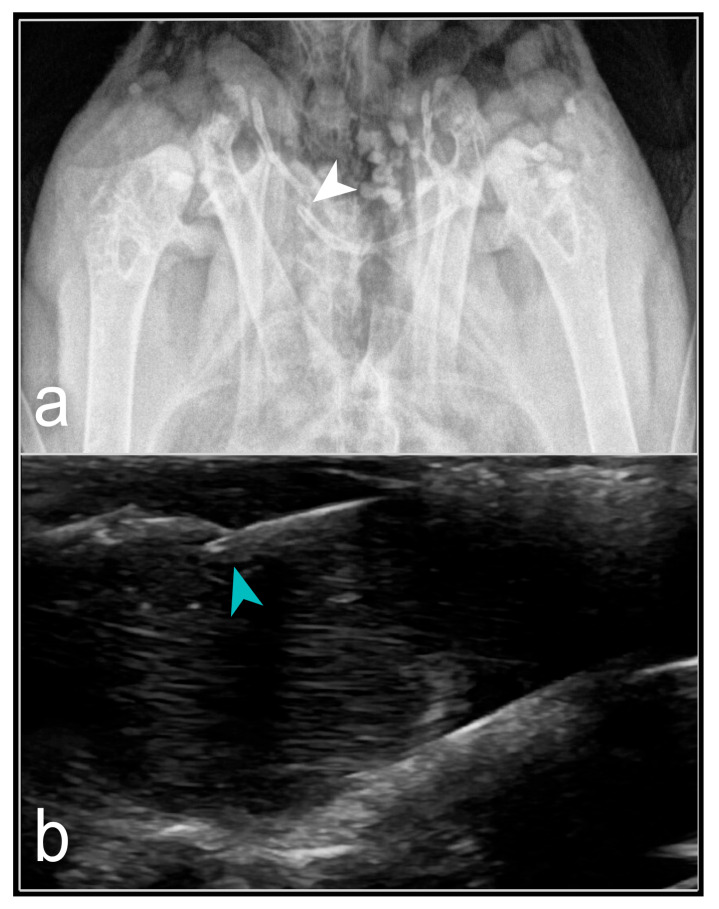
Displaced oblique fracture of the clavicle (*Os clavicula*) in a feral pigeon (*Columba livia* f. domestica): (**a**) shows the fracture in a ventrodorsal RX (white arrowhead), whereas (**b**) shows the fracture in a B-mode US, longitudinal section, craniocaudal approach, ventrodorsal plane, ‘hockey stick’ transducer, 15 MHz (blue arrowhead). Source: A. Korshunova.

Fractures predominantly occurred in the shoulder girdle, particularly in the clavicle (26.3%), the coracoid (5.6%), and the scapula (2.6%). The fracture area revealed two vertically dislocated bone fragments (Figure 1). No soft tissue swelling or haemorrhage (hematoma) was commonly present. The superimposition of bone and soft-tissue structures that form the shoulder girdle in birds frequently prevented the visualisation of clavicular disorders on conventional RX (ventrodorsal and laterolateral). Ultrasound imaging was challenging due to the clavicle’s proximity to the skin and the absence of soft tissue in the subcutaneous layer, resulting in an inadequate buffer zone between the ultrasound probe and the clavicle.

**Figure 2:**
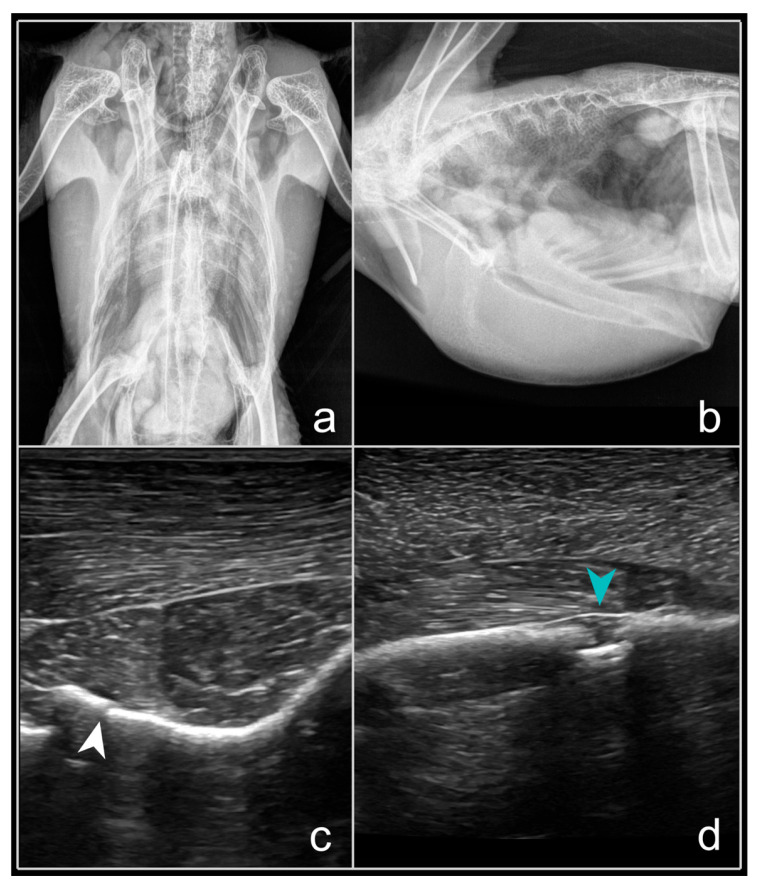
Compounded fracture of the sternum (*Os sterni*), middle region of the sternal body (*Corpus sterni*), in an African grey parrot (*Psittacus erithacus*). (**a**)—ventrodorsal RX reveals unclear pathology; (**b**)—laterolateral RX also demonstrates unclear pathology; (**c**)—fracture displayed in US image, B-mode, cross-section, ventral access, ‘hockey stick’ transducer, 15 MHz (white arrowhead); (**d**)—fracture displayed in US image, B-mode, longitudinal section, ventral access (blue arrowhead), fracture location identifiable as a hypoechoic region, with a bone fragment (hyperechoic structure) beneath, ‘hockey stick’ transducer, 15 MHz. Source: A. Korshunova.

The fracture site was typically visualised as a small, hypoechoic area that disrupted the hyperechoic line of the sternum both longitudinally and transversely in the diagnosis of sternum and keel fractures (Figure 2). Significant swelling of the muscular tissue was observed, characterised by a ‘blurring’ of the muscle fibre architecture in the ultrasound image and by haemorrhaging. The lack of distinctive clinical signs complicated overall identification of this ailment, especially the inability to fly, often without wing droop, and a history of suspected injury; a haematoma in the sternum region was identified in only one-third of cases. In one-third of cases, the sternum’s structure was intact on the RX.

### 3.2. Long Peripheric Bones

#### 3.2.1. Common Fractures

In diagnosing fractures of long peripheral bones, radiology was more effective than US. Sonographic examination typically demonstrated two or more bone fragments, frequently displaced either vertically or horizontally in comminuted fractures. Moreover, considerable edema of the muscular tissue and haemorrhaging were typically noted. US was especially effective in assessing ‘old’ fractures during the callus development phase.

#### 3.2.2. Pathological Fractures

**Figure 3:**
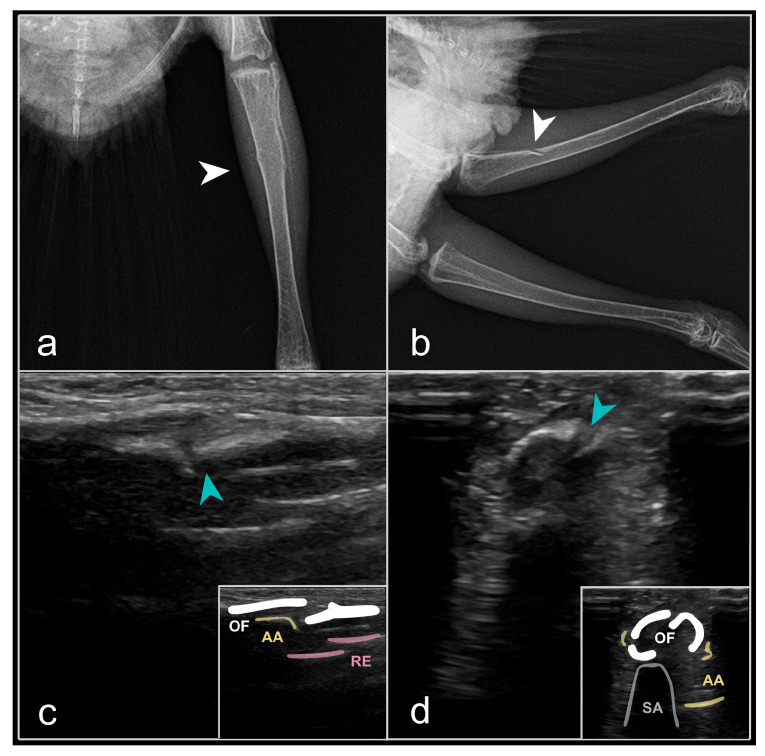
Pathological fracture (‘greenstick fracture’) of the tibial body (*Corpus tibiae*), located in the middle section of the tibiotarsus, proximal third of the bone, in a feral pigeon (*Columba livia* f. domestica). (**a**)—ventrodorsal RX, with the fracture site appearing as a slightly indistinct “stripe” (white arrowhead); (**b**)—laterolateral RX, with the fracture site visible as a “fissure” in the bone cortex (*Substantia compacta*, cortex) (white arrowhead); (**c**)—US image depicting the fracture as a hypoechoic area between the bone fragments, longitudinal section, medial access, ‘hockey stick’ transducer, 15 MHz (blue arrowhead); (**d**)—US image depicting the fracture as a hypoechoic area (‘fissure’) between the bone fragments, B-mode, cross-section, medial access, ‘hockey stick’ transducer, 15 MHz (blue arrowhead). Legends: OF—osseous fragments, AA—auditory amplification (acoustic artefact), RE—recurrent echoes (acoustic artefact), SA—shadow artefact (acoustic artefact). Source: A. Korshunova.

The US characteristics of pathological fractures, including tibial and radial fractures, exhibited a partial disruption of the periosteal integrity of the damaged bone, manifesting as a hypoechoic ‘tear’ without movement of the bone fragments (Figure 3). No soft tissue swelling or hemorrhage was observed in this condition. The primary clinical signs included: dysfunction of the affected limb and limping (in instances of tibial injuries). The radiographic diagnosis was complex, as the fracture was confined to a restricted area and the bone pieces remained non-displaced.

### 3.3. Knee Arthrosis

US did not clearly identify osteoarthritis of the knee joint, making a detailed evaluation of its ultrasonic structure unfeasible. An irregular structure of considerable echogenicity in the joint space between the femur and tibia could be observed. Healthy birds exceeding 300 g exhibit a small, hypoechoic joint gap between the femur and tibia. Radiographically, this condition was characterised by clear hyperplastic changes in the articular cartilage.

### 3.4. Bone Healing Monitoring

**Figure 4:**
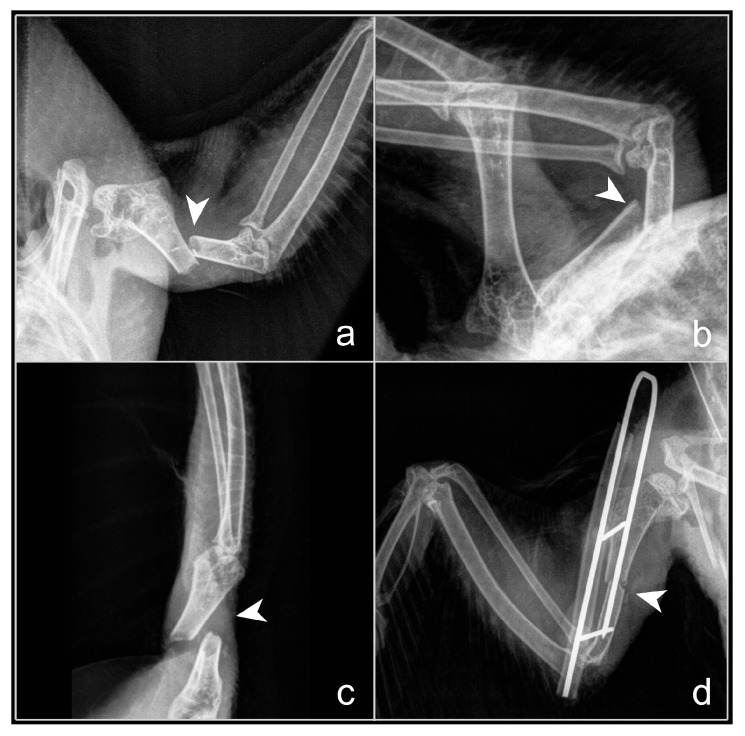
Displaced oblique fracture of the humerus (*Os humerus*) in a feral pigeon (*Columba livia* f. domestica), RX representation of the fracture (white arrowhead): (**a**)—ventrodorsal plane; (**b**)—laterolateral plane; (**c**)—craniocaudal plane; (**d**)—ESF tie-in bone fixation, ventrodorsal plane. Source: A. Korshunova.

US assessment demonstrated clinical relevance for assessing bone healing in patients who underwent surgical intervention for fractures of hollow peripheral bones using the ESF tie-in technique (Figure 4). US revealed the development of secondary cartilage at the fracture site on days 7 to 8 post-operatively.

**Figure 5:**
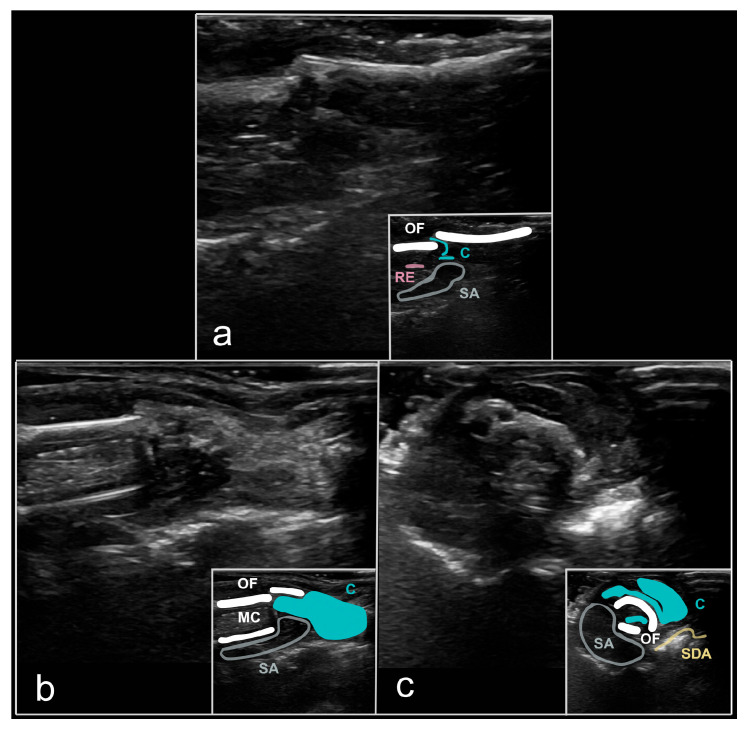
Displaced oblique fracture of the humerus (*Os humerus*) in a feral pigeon (*Columba livia* f. domestica), monitoring bone healing post-surgical fixation (ESF tie-in) using B-mode US, ‘hockey stick’ transducer, 15 MHz. (**a**)—7 days post-surgery, longitudinal section, medioventral access, minimal callus formation observed; (**b**)—14 days post-surgery, longitudinal section, medioventral access, pronounced callus visible; (**c**)—14 days post-surgery, cross-section, medioventral access, pronounced callus visible. Legends: OF—osseous fragments, C—callus (fracture callus), MC—medullary cavity, SDA—sound amplification (acoustic artefact), RE—reverberating echoes (acoustic artefact), SA—shadow artefact (acoustic artefact). Source: A. Korshunova.

The cartilage manifested as an uneven, indistinct mass exhibiting reduced echogenicity compared to the bone (Figure 5). Between days 10 and 14, it was primarily noted that the bone tissue (callus) extended beyond the limits of the periosteum of the bone, enclosing the fracture site. Unlike the ultrasonic study, the development of secondary cartilage was observable on RX images between days 10 and 14.

### 3.5. Tendinitis of the Shoulder Joint

**Figure 6:**
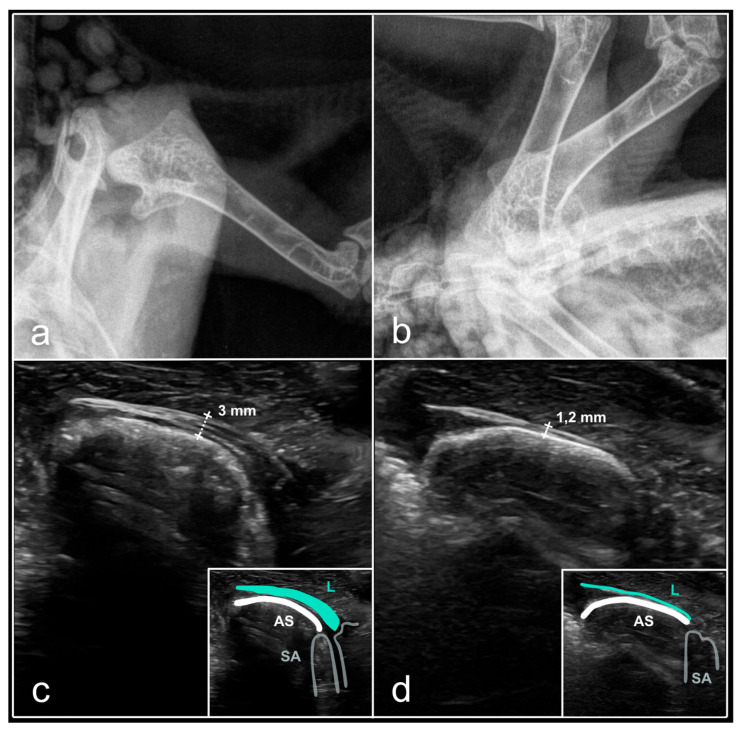
Tendinitis of the shoulder joint (*Articulatio humeri*) in the feral pigeon (*Columba livia* f. domestica). (**a**)—ventrodorsal X-ray, pathology not evident; (**b**)—laterolateral X-ray, pathology not evident; (**c**)—B-mode ultrasound, longitudinal section, craniodorsal access, the joint surface of the humeral head (*Caput humeri*) appears irregular, the acrocoracohumeral tendon (*Ligamentum acrocoracohumeralis*) exhibits a ‘beak-shaped’ morphology and is markedly thickened (3 mm in the central region) ‘hockey stick’ transducer, 15 MHz; (**d**)—B-mode ultrasound image of the intact shoulder joint, longitudinal section, craniodorsal access, the joint surface of the humeral head (*Caput humeri*) appears as a hyperechoic flat structure, the acrocoracohumeral tendon measures 1.2 mm in thickness in the central region, ‘hockey stick’ transducer, 15 MHz. Legends: AS—articular surface of the humeral head (*C. humeri*), L—acrocoracohumeral tendon (*L. acrocoracohumeralis*), SA—shadow artefact (acoustic artefact). Source: A. Korshunova.

In two birds, inflammation of the shoulder joint tendon was detected exclusively by ultrasound. The demonstrated clinical signs included functional impairment of the injured wing without drooping, inability to fly, and pain upon rotation of the injured joint, with no signs of crepitus. These cases presented as significant tremors or marked motor activity in the immobilised patient during orthopaedic assessments. Ultrasound revealed the following findings: thickening of the tendon beneath the coracoid process (*Ligamentum acrocoracohumerale*), an uneven surface of the humeral head (*Caput humeri*) with localised calcifications, and an unaltered muscle layer (Figure 6). Radiological assessment indicated no anomalies in these cases.

### 3.6. Luxation of the Shoulder Joint

A dislocated shoulder joint was diagnosed in one feral pigeon. The ultrasound findings indicated significant displacement of the osseous tissues, complicating visualisation of the entire joint. Evidence of injury to the integrity of the joint capsule and edema of the adjacent soft tissues was also present.

### 3.7. Data Analysis

#### 3.7.1. Sensitivity and Specificity

Radiological examination demonstrated a specificity of 85.7% and a sensitivity of 70.7% in the entire cohort. The positive predictive value was 93.5%, whilst the negative predictive value was 50.0%. Ultrasound examination revealed a substantially higher sensitivity of 97.6% in the entire group. The specificity was absolute, at 100%. The positive predictive value was 100%, while the negative predictive value was 93.3%. In the fracture subgroup, RX analysis demonstrated a sensitivity of 75.7% and a specificity of 85.7%. The positive predictive value was 93.3%, whereas the negative predictive value was 57.1%. In the fracture subgroup, US had a sensitivity of 97.3% and a specificity of 100% and showed positive predictive value at 100% and negative predictive value at 93.3%.

#### 3.7.2. Concordance of the Diagnostic Methods

The total group exhibited a Kappa value of κ = 0.64, indicating considerable agreement (z = 4.94; *p* = 7.65 × 10^−7^). In the fractures subgroup, the Kappa score was κ = 0.737, indicating substantial to good agreement (z = 5.35; *p* = 8.95 × 10^−8^).

#### 3.7.3. Comparison of Representability and Interpretability via Fisher’s Exact Test

The whole group showed *p*-values of 0.513 for visualisation and 1.0 for interpretability, indicating no statistically significant difference between the two diagnostic imaging tools. In the fracture subgroup, *p* = 0.637 for visualisation and *p* = 0.748 for interpretability, indicating no significant differences between RX and US assessments.

## 4. Discussion

In the present study, RX and US examinations were systematically compared in birds with suspected orthopaedic conditions. Statistical analyses were conducted independently for the entire cohort and the fracture subgroup, employing both descriptive and inferential techniques. In both groups, US demonstrated higher sensitivity than radiography, indicating an increased detection rate of the investigated pathologies within the context of this study. The US also demonstrated high specificity in both cohorts, while radiography showed comparatively lower specificity. The positive predictive values were high for both modalities, reaching 100% for ultrasonography in both the overall and fracture groups. In addition, the negative predictive values for ultrasonography were higher than those for radiography, suggesting that a negative sonographic finding may reliably indicate the absence of pathology under the conditions of this study. The analysis of diagnostic agreement using Cohen’s kappa revealed substantial to good concordance between imaging findings and the comprehensive clinical evaluation in both cohorts [27,28], with slightly higher agreement observed in the fracture subgroup. No statistically significant differences were found between radiography and ultrasonography for image presentation and interpretability in either cohort. As US examinations were performed after RX assessment, prior knowledge of radiographic findings may have influenced the interpretation of ultrasound images, potentially increasing the apparent sensitivity of ultrasonography by facilitating targeted evaluation of suspected lesions. Conversely, this approach may also have contributed to an overestimation of specificity, as equivocal findings could have been interpreted in light of previously identified radiographic results. Therefore, the observed differences in diagnostic performance between modalities should be interpreted with caution due to the non-blinded, sequential study design.

The study by Gonzalez et al. (2018) delineated the utility of US examination for evaluating the periosteal status of the hollow bones in the peripheral skeleton and for real-time visual monitoring during orthopaedic surgical interventions [14]. Our previous research also identified prospective applications of this technique for visualising osseous and selected soft tissue features of the MSK system in feral pigeons [23]. The study also highlighted the potential for visualising a comprehensive cross-section of hollow bones in avian species weighing between 300 and 1000 g [23]. MSK ultrasonography is gaining prominence in small animal medicine. Cook (2016) delineates the diagnostic options for osteomyelitis in canines, which is characterised by an uneven, hyperechoic formation on the bone surface [29]. Analogous alterations have been noted in avians exhibiting shoulder joint tendinitis, suggesting an inflammatory element that may similarly impact the osseous architecture of the humeral head. The author emphasises the method’s potential for monitoring bone healing, aligning with our study’s findings. The results of this study show that ultrasonic examination can be used to monitor orthopaedic patients after surgery, which allows for the quick removal of surgical fixation and encourages the best possible recovery.

Ultrasound has been recognised as an effective technique for diagnosing diseases of ligamentous structures and joints in canines and felines [18,21]. Normal ligaments are defined as slender structures featuring parallel hyperechoic fibres, while tendinopathies or tendinitis generally present as thickening of the ligamentous structure, frequently associated with fluid accumulation within the ligamentous sheath, and may occasionally involve localised mineralisation of the tissue [18,29,30]. Our observations indicate that tendinitis in two birds presented exclusively as thickening of the ligamentous structure, with no observable synovial fluid accumulation, possibly due to the structural features of the ligamentous apparatus in birds [31]. Shoulder joint disorders (tendinitis, tenosynovitis, ligament tears, myositis) are prevalent in dogs. US examination involves assessing the anatomy of the supraspinatus, infraspinatus, and biceps tendons/muscles, which may be constrained by the challenging access to the medial shoulder joint [18]. However, its diagnostic utility may be limited by restricted acoustic access to deeper or medially located joint structures [18]. Consequently, certain lesions of the medial shoulder compartment may not be completely visualised, which may necessitate complementary imaging modalities in selected cases.

Our findings indicate that the ultrasound picture of the avian shoulder joint exhibits notable changes compared to those documented in canines and felines. We hypothesise that the observed variation is associated with the markedly diminished musculature responsible for shoulder joint functionality in birds, along with the distinctive anatomical configuration of the shoulder, comprising three joints of bony structures [31]. The limited number of tendinitis cases (*n* = 2) restricts the strength of conclusions, and these findings should be interpreted with caution. The diagnosis of knee arthrosis was established in only one patient, and imaging was limited; thus, our data are inadequate to definitively assess the efficacy of ultrasound scanning for diagnosing knee joint arthropathies. The findings of the present study suggest that ultrasound may be a supplementary approach for assessing the long bones, sternum, shoulder girdle, and shoulder area in birds, as well as for evaluating injury age and prognosis, and for determining therapy options.

Although ultrasonography offers a broad range of applications, species-specific factors may limit its diagnostic utility in birds. In particular, reverberation artefacts caused by gas within pneumatised bones can impair image quality and hinder assessment of deeper osseous structures [32,33]. In addition, the superficial location and minimal soft tissue coverage of certain anatomical regions, such as the clavicle, may complicate probe positioning and lead to artefacts (e.g., mirror effects), thereby reducing image interpretability. The MSK ultrasonography in small animals has also been described as subject to various limitations, including physical artefacts and operator dependency [33,34,35]. These limitations highlight the importance of operator experience and careful image interpretation when performing MSK ultrasound in avian patients.

This study design also has several limitations, including a small sample size and a limited range of disorders examined. A further limitation of this study is the uneven species distribution, with a predominance of pigeons (*n* = 29, out of a total of 55), which may restrict the generalisability of the findings to other avian species. In addition, most birds included in this study fell within a moderate body weight range, and therefore, the applicability of the results to very small (<130 g) or large (>3000 g) species remains limited. The findings are most directly relevant to birds weighing approximately 200–1000 g. Furthermore, the absence of a post-mortem examination to obtain a pathological evaluation in the present study may limit the relevance of the clinical results in relation to the pathological findings. Due to the lack of blinding between imaging modalities, the statistical findings should be interpreted with caution and are operator-dependent. The study design was intentionally chosen to reflect clinical workflow conditions, in which imaging modalities are typically applied sequentially rather than independently. However, this approach may have influenced the diagnostic performance of US and should be considered when interpreting the results. This highlights the need for further research employing blinded or randomised designs, focusing on the specificity and sensitivity of US examinations across a broader spectrum, accounting for a range of avian species, anatomical features, and categories of MSK disorders.

## 5. Conclusions

The results of this study indicate that B-mode ultrasonography is a valuable supplementary imaging technique for assessing avian orthopaedic disorders, while radiography remains the primary method for assessing skeletal structures, particularly fractures. This finding should be interpreted with caution due to the non-blinded, sequential study design. Despite various limitations, this technique may be especially beneficial in addition to RX in cases of inconclusive or negative radiographic findings, in the presence of clinical signs suggesting localised pain or MSK dysfunction, in cases of suspected pathological fractures of peripheral bones or fractures of the sternum, and potentially in cases of suspected inflammatory processes of the shoulder joint. Moreover, US may be useful for monitoring callus formation during fracture healing in birds weighing 200–1000 g. Further studies using blinded or randomised designs with a larger and more varied sample are warranted to validate these findings.

## Figures and Tables

**Table 1:** Data on the clinical signs of birds presented in the study.

Clinical Signs	Clinical Cases	
	*n*	%
Flying inability	30	54.5
Lameness	8	14.5
Hanging wing	3	5.5
Torticollis	1	1.8
Skin wound, torticollis	1	1.8
Lameness, apathy	3	5.5
Skin wound, hanging wing	2	3.6
Wet breathing sounds	3	5.5
Knee stiffness	1	1.8
Torticollis, hanging wing	3	5.5
Total	55	100

**Table 2:** Bird species included in the study with body weight data (total *n* = 55).

Species	Body Weight in g (Mean ± SD)	Number, *n*
Feral pigeon (*Columba livia* f. dom)	257.35 ± 30.95	29
Common wood pigeon (*Columba palumbus*)	343.83 ± 46.76	8
Eurasian collared dove (*Streptopelia decaocto*)	130.0	1
Common kestrel (*Falco tinnunculus*)	154.33 ± 26.76	3
Red kite (*Milvus milvus*)	390.0	2
Common buzzard (*Buteo buteo*)	706.0	4
African grey parrot (*Psittacus erithacus*)	480.0	1
European green woodpecker (*Picus viridis*)	130.0	1
White stork (*Ciconia ciconia*)	2996.66 ± 15.27	3
White cockatoo (*Cacatua alba*)	450.0	1
Carrion crow (*Corvus corone*)	450.0	1
Eurasian jay (*Garrulus glandarius*)	187.0	1

SD, standard deviation.

**Table 3:** A comprehensive overview of diagnoses and their confirmation by X-ray and ultrasound examinations.

Diagnosis	Total		Confirmed by X-Ray		Confirmed by US	
	*n*	% ^1^	*n*	% ^2^	*n*	% ^2^
Fracture	28	50.9	24	85.7	27	96.4
Pathological fracture	3	5.5	1	33.3	3	100.0
Tendinitis	2	3.6	2	100.0	2	100.0
Luxation	1	1.8	1	100.0	1	100.0
Arthrosis	1	1.8	1	100.0	1	100.0
Healing fracture	6	10.9	3	50.0	6	100.0
Intact	14	25.5	12	85.7	14	100.0

^1^ The respective percentage of the cases confirmed by each modality based on the absolute case numbers; ^2^ The respective percentage of the cases confirmed by each modality based on the corresponding category case numbers.

## Data Availability

The raw data supporting the conclusions of this article will be made available by the authors on request.

## Abbreviations

The following abbreviations are used in this manuscript:

UVB	Ultraviolet B radiation
MBD	Metabolic bone disease
RX	Radiography, radiographic
CT	Computed tomography
US	Ultrasound, ultrasonography
MSK	Musculoskeletal
PO	*Per os*, orally
BID	*Bis in die*, twice a day
ESF	External skeletal fixation
IMP	Intramedullary pin
SD	Standard deviation
OF	Osseous fragments
AA	Auditory amplification
RE	Recurrent echoes
SA	Shadow artefact
MC	Medullary cavity
SDA	Sound amplification
AS	Articular surface of the humeral head
L	Acrocoracohumeral tendon
